# Interferon Beta-1a (AVONEX^®^) as a Treatment Option for Untreated Patients with Multiple Sclerosis (AXIOM): A Prospective, Observational Study

**DOI:** 10.3390/ijms160715271

**Published:** 2015-07-06

**Authors:** Christoph Kleinschnitz, Gabriele Niemczyk, Karin Rehberg-Weber, Colin Wernsdörfer

**Affiliations:** 1 Department of Neurology, University Hospital Würzburg, Würzburg D-97070, Germany; 2 Biogen Idec GmbH, Ismaning D-85737, Germany; E-Mails: Gabriele.Niemczyk@biogen.com (G.N.); karin.rehberg-weber@biogen.com (K.R.-W.); colin.wernsdoerfer@biogen.com (C.W.)

**Keywords:** relapsing-remitting multiple sclerosis, disease-modifying therapy, injection site reactions, quality of life, efficacy

## Abstract

The efficacy and safety of first-line disease-modifying therapies (DMT) for relapsing-remitting multiple sclerosis (RRMS) has been demonstrated in pivotal, randomized trials, but these studies do not reflect the routine care setting where treatment gaps or switches are common. The Avonex as Treatment Option for Untreated MS Patients (AXIOM) trial assessed the efficacy of newly-initiated intramuscular interferon beta-1a (IM IFNb-1a) after a treatment-free interval, with particular consideration of the previous course of disease and therapy. The AXIOM trial was an open, 12-month, observational, non-interventional study with a retrospective and a prospective part conducted in Germany. RRMS patients with a treatment-free interval of at least three months were included and treated with IFNb-1a for up to 12 months. Relapse rate, disability progression, injection-related parameters and quality of life observed during the prospective part were compared with retrospectively-collected data. Two hundred and thirty five RRMS patients participated in AXIOM. The mean relapse rate decreased from 1.1 in the three months before baseline to 0.2 per quarter during the twelve-month observational period; the Multiple Sclerosis Functional Composite score improved during twelve months of IM IFNb-1a treatment, while the Expanded Disability Status Scale score did not change over the course of this study. Compared to previous DMTs (IM IFNb-1a, subcutaneous IFNb-1a (SC IFNb-1a), SC IFNb-1b, glatiramer acetate), the patients experienced less injection site reactions and flu-like symptoms, with a stated improved quality of life. IM IFNb-1a was effective and well accepted in RRMS patients with no or discontinued previous therapy. These results from the routine care setting may inform optimization of DMT treatment in RRMS, but need confirmation in further studies.

## 1. Introduction

Intramuscular interferon beta-1a (IM IFNb-1a, Avonex^®^), subcutaneous IFNb-1a (SC IFNb-1a, Rebif^®^), subcutaneous IFNb-1b (SC IFN-beta-1b, Betaferon^®^, Extavia^®^) and glatiramer acetate (GA, Copaxone^®^) are approved first-line disease-modifying therapies (DMT) of relapsing-remitting multiple sclerosis (RRMS). Each of the approved IFNb preparations for relapsing forms of MS has demonstrated efficacy as measured by reduced relapse rates, delayed progression of disability and reduced number of lesions detected by magnetic resonance imaging (MRI) in pivotal phase III clinical trials [1].

Due to factors, such as strict patient selection, adhesion to study protocol and close monitoring of patients’ neurological status, clinical trials do not adequately reflect everyday clinical practice. This includes delayed treatment initiation, treatment interruptions and switching between DMTs. Treatment gaps or early discontinuation of DMT treatment may adversely affect the clinical disease course in MS patients. Data from a national managed care database in 2388 MS patients treated with DMTs disclosed a nearly two-fold increase in the risk of severe relapses in patients with treatment gaps of 90 days or longer compared to intermissions of only 0–10 days [2]. In a study of patients with high disease activity prior to treatment initiation and good response to IFNb therapy, disease activity rapidly returned to the previous level after discontinuation of IFNb, and every fifth patient experienced a relapse within 30 days [3].

The effects of previous DMT treatments, treatment changes or treatment-free intervals on the efficacy of subsequent DMT treatment, the motivation to change DMT therapy and patient satisfaction following a switch of DMT are insufficiently characterized. Such data would be of high clinical relevance to optimize routine DMT treatment. Accordingly, the identification of potential candidates for DMT treatment adjustment or the knowledge of factors associated with treatment persistence has been considered an important issue for treatment optimization [4,5,6].

The Canadian Multiple Sclerosis Working Group (CMSWG) has developed practical recommendations based on objective clinical criteria (attacks, disability progression and MRI) to help physicians to optimize therapy [7]. However, more definitive consensus criteria for the routine clinical management of RRMS patients receiving DMTs have been demanded [8], and no universal guidelines on switching DMTs are available [9]. The published switch criteria, such as those of the CMSWG, are largely focused on treatment failure [7], but other factors, such as adverse experiences or poor patient acceptance of the injection regimen, may warrant a treatment modification, even in patients with good treatment response.

The primary objective of the non-interventional, observational AXIOM trial (Avonex as Treatment Option for Untreated MS Patients) was to assess the efficacy of IM IFNb-1a after a treatment-free interval of at least three months under routine care conditions in a cohort of German RRMS patients. A secondary objective was to collect retrospective data on the disease and treatment history, which may help neurologists to optimize therapy with first-line DMTs.

## 2. Results

### 2.1. Subjects

In total, 235 patients were recruited, and all were included in the analysis. The number of patients with analyzable data was 235 at Visit 1 (baseline), 168 at Visit 2, 148 at Visit 3, 129 at Visit 4 and 107 at Visit 5. Data from 103 patients were available at all visits. The mean observation period was 222.1 days (±162.1, standard deviation). Most of the patients were female (68.5%), and 28.9% were male (*n* = 6 missing values). The participants’ average age was 38.6 (±10.5) years (*n* = 230/235, the patient numbers refer to those with evaluable data for the respective endpoint). The average body mass index of the adult male (25.0 kg/m^2^, 60/66) and female patients (24.1 kg/m^2^, 149/159) was in the upper normal range. The mean time since diagnosis of MS was 6.3 years (range: 0.3 to 405.5 months; 225/235). The most frequent first symptoms of MS were dysesthesia or hypesthesia (49.8%) and visual disturbances (44.7%, *n* = 9 missing).

### 2.2. Previous MS-Specific Treatment (Retrospective Part)

**Figure 1:**
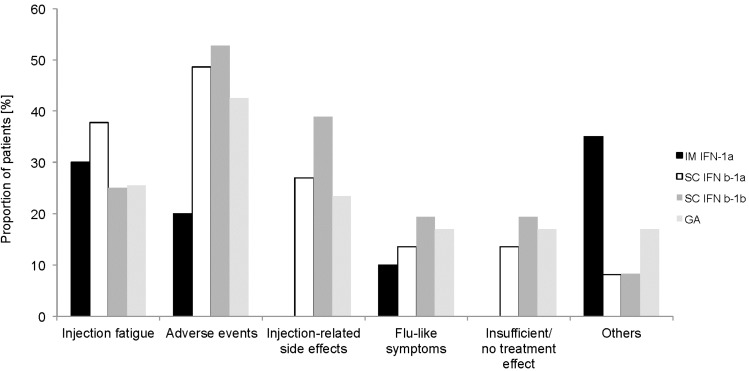
Reasons for stopping previous MS-specific treatment stratified by type of disease-modifying therapy (DMT) (multiple entries permitted). IM IFNb-1a, intramuscular interferon beta-1; SC, subcutaneous; GA, glatiramer acetate.

For a total of 107 of 235 patients (45.5%), no information for previous therapy was available. GA was the most commonly-used previous MS treatment (20.0%), followed by SC IFN beta-1a (15.7%) and SC IFN beta-1b (15.3%), relative to the total patient sample (*n* = 235, multiple entries possible). Comparatively fewer patients reported previous treatment with IM IFNb-1a (8.5%), natalizumab (3.4%) or others (7.2%). Multiple entries were permitted. Due to the small numbers of patients pre-treated with natalizumab (*n* = 8) or other medications, no further data on these patients are reported. The average time between first diagnosis of MS and initiation of MS therapy was 37.5 ± 65.1 months (*n* = 120/235). The mean duration of previous MS-specific treatment was shortest for GA (20.4 months) and longest for IM IFNb-1a (35.7 months). Injection fatigue was a common reason to discontinue previous MS-specific treatment, largely independent of the formulation used (mean percentages between 25.0% and 37.8%). Side effects (42.6% to 52.8%) were more frequently reported as reasons to discontinue the previous SC formulations (*n* = 37 IFN beta-1a, *n* = 36 IFN beta-1b, *n* = 47 GA) compared to IM IFN beta-1a (20.0%, *n* = 20). A considerable proportion of patients stopped treatment with the SC formulations due to injection-related side effects (23.4% to 38.9%), while none of the patients previously treated with IM IFN beta-1a mentioned injection-related side effects as a reason for treatment cessation. Flu-like symptoms as a reason to discontinue prior treatment were only reported for the IFNb formulations (10.0% IM IFNb-1a to 19.4% SC IFNb-1b), but not for GA. Insufficient or absent efficacy was a reason to discontinue the previous SC medications (13.5% SC IFNb-1a to 19.4% SC IFNb-1b), but not IM IFNb-1a (Figure 1).

### 2.3. Treatment with IM IFNb-1a (Prospective Part)

**Figure 2:**
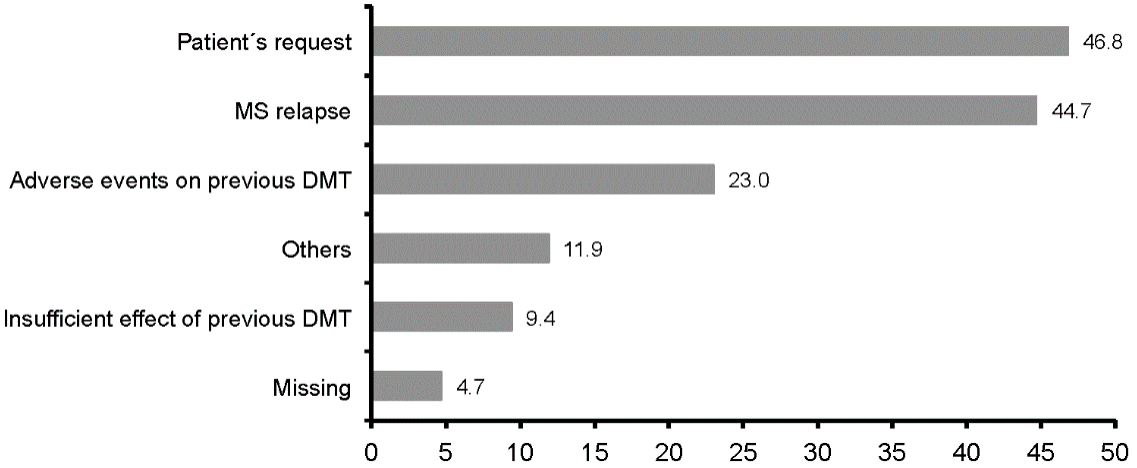
Reasons for initiating treatment with IM IFNb-1a (multiple entries permitted, *n* = 235). Patient’s wish: *n* = 110/235; MS relapse: *n* = 105/235; adverse events during previous therapy: *n* = 54/235; other: *n* = 28/235; insufficient or absent efficacy of previous therapy: *n* = 22/235; missing: *n* = 11/235.

The interval between discontinuation of the previous MS treatment and start of prospective treatment with IM IFNb-1a ranged between 13.5 ± 22.3 months (GA) and 33.8 ± 32.2 months (IM IFNb-1a) on average (Table 1). In rare cases, the time period between the end of the previous MS therapy and the initiation of IFNb-1a was shorter than three months or patients were immediately switched to IFNb-1a. The most common reasons to initiate treatment with IM IFNb-1a were patient’s wish (46.8%, *n* = 110/235) and occurrence of an MS relapse (44.7%, *n* = 105/235), followed by side effects of previous therapy (23.0%, *n* = 54/235; Figure 2).

**Table 1:** Time interval (months) between discontinuation of previous MS-specific treatment and Visit 1.

Value	IM IFNb-1a (*n* = 15/20) ^1^	SC IFNb-1a (*n* = 33/37) ^1^	SC IFNB-1b (*n* = 32/36) ^1^	GA (*n* = 44/46) ^1^	Natalizumab (*n* = 6/8) ^1^
Mean (SD)	33.8 (32.2)	23.0 (31.5)	26.1 (36.5)	13.5 (22.3)	14.8 (12.1)
95% CI	16.0–51.7	11.8–34.1	13.0–39.3	6.8–20.3	2.0–27.5
Range	2.5–103.2	0.0–112.2	0.0–171.5	0.2–94.6	1.5–31.9

CI, confidence interval; ^1^ the number of evaluable values (plausible, not missing) of total patients with data about pre-treatment.

**Figure 3:**
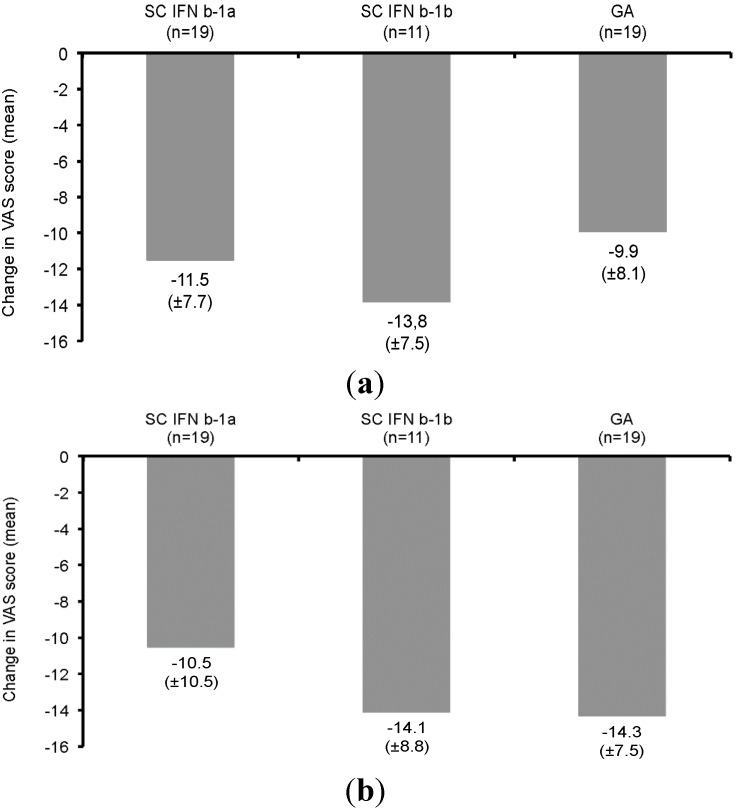

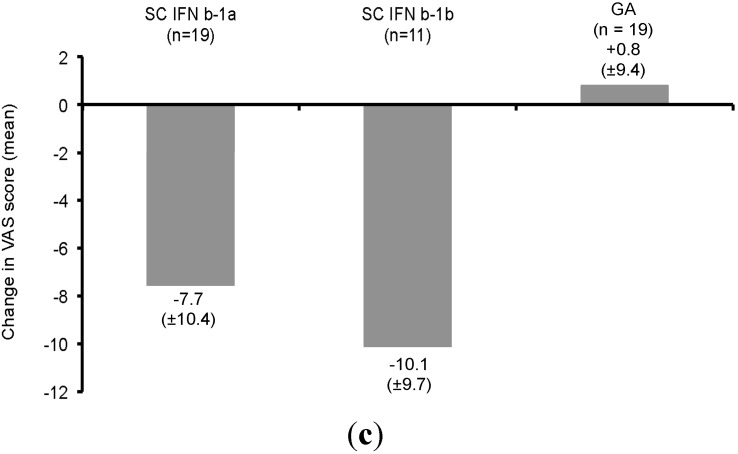
Mean difference (±SD) of the magnitude of injection fatigue (**a**), injection-related reactions (**b**) and flu-like symptoms (**c**) between current IM IFNb-1a and previous MS treatments at six months as rated by patients on a Visual Analog Scale (VAS) (1 = none to 25 = very intense, negative values indicate improvement).

At Visit 3, about half a year after the start of prospective IM IFNb-1a treatment, patients were asked to rate the magnitude of injection fatigue, injection side effects and flu-like symptoms of current therapy in comparison to the previous MS-specific therapy on the VAS (1 = none to 25 = very intense). Only relatively few data were available for these parameters (*n* = 2 IM IFNb-1a, *n* = 19 SC IFNb-1a, *n* = 11 SC IFNb-1b and *n* = 19 GA). As only data from two patients who had previously received IM IFNb-1a were documented, these were considered not interpretable (data not shown, ditto flu-like symptoms and quality of life). In comparison to the previous SC formulations (thrice-weekly, or every-other-day IFNb, or once-daily GA), both self-reported injection fatigue (*p* = 0.4319) and injection-related side effects (*p* = 0.3748) were numerically lower on current IM IFNb-1a than on previous treatments (indicated by negative values; Figure 3).

**Figure 4:**
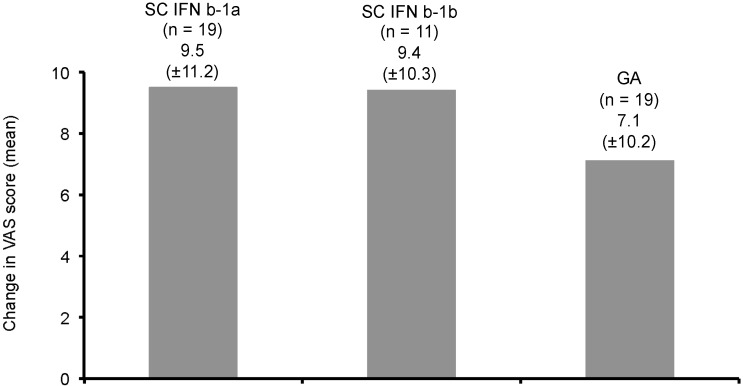
Mean difference of quality of life between current IM IFNb-1a and previous MS-treatments at six months as rated by the patients on a VAS (1 = poorest to 25 = best, positive values indicate improvement).

At Visit 3, patients rated flu-like symptoms on two separate Visual Analog Scales for current IM IFNb-1a treatment and previous treatments, respectively. Flu-like symptoms were significantly less common on current IM IFNb-1a treatment than on previous treatment (*p* = 0.0055). The differences in favor of current IM IFNb-1a were most pronounced compared to SC IFNb-1a (−7.7 ± 10.4, a negative value indicates better rating) and SC IFNb-1b (−10.1 ± 7.9), while the magnitude of flu-like symptoms was overall comparable between current IM IFNb-1a and previous GA (0.8 ± 9.4). The comparison of the patient’s ratings of quality of life on the VAS (1 = poorest to 25 = best) showed an advantage of current IM IFNb-1a compared to prior treatments (*p* = 0.7423). The improvement of quality of life on current therapy *vs.* SC IFNb-1a (9.5 ± 11.2, a positive value indicates improvement) and SC IFNb-1b (9.4 ± 10.3) was of similar magnitude, while the difference in comparison to GA was slightly less pronounced (7.1 ± 10.2; Figure 4).

As shown in Table 2, the average number of relapses in the three months preceding treatment with IM IFNb-1a, when the majority of patients did not receive MS-specific treatment, was 1.1 ± 0.3 (*n* = 123), of which most were treated with steroids (1.0 ± 0.4, *n* = 117). In the last 12 months prior to study entry, the number of relapses was 1.8 ± 1.4 (*n* = 177), and on average, 1.3 ± 0.9 relapses had required steroid treatment. During the prospective phase, the number of relapses per quarter was lower than in the last three months before initiation of current treatment. The relapse rate remained stable (0.2 per quarter on average) over 12 months of prospective treatment with IM IFNb-1a. The mean rate of steroid-treated relapses per quarter during the 12-month therapy with IM IFNb-1a (mean values between 0.3 and 0.4 per quarter) was apparently higher than the total relapse rate. Plausible data on steroid-treated relapses were only reported for a few patients (between 32 and 55 per quarter), and these data likely refer to a subgroup of patients with probably more severe relapses, which might explain the discrepancy between the rate of total and steroid-treated relapses [10].

**Table 2:** Number of relapses during the twelve months and the three-month periods prior to study baseline (retrospective) and in the 3, 6, 9 and 12 months after the start of IM IFNb-1a treatment (prospective).

Value	Number of Relapses per 3 Months of Time					
	Retrospective Observational Period Prior to Study Entry		Prospective Observational Period after Study Entry			
	12 Months Preceding Baseline (*n* = 177)	3 Months Preceding Baseline (*n* = 123)	3 Months Preceding Visit 2 (*n* = 158/168) ^1^	3 Months Preceding Visit 3 (*n* = 142/148) ^1^	3 Months Preceding Visit 4 (*n* = 123/129) ^1^	3 Months Preceding Visit 5 (*n* = 103/107) ^1^
Mean (SD)	0.45 (0.35)	1.1 (0.3)	0.1 (0.4)	0.2 (0.4)	0.2 (0.4)	0.1 (0.4)
95% CI	0.4–0.5	1.0–1.1	0.1–0.2	0.1–0.2	0.1–0.2	0.1–0.2
Range	0.25–3.0	1.0–2.0	0.0–2.0	0.0–2.0	0.0–1.0	0.0–2.0

^1^ The number of evaluable values (plausible, not missing) of total patients available at the respective follow-up visit.

The available data showed that the mean EDSS was 1.9 ± 1.5 twelve months (*n* = 116) and 2.0 ± 1.4 three months (*n* = 160) prior to study entry. The EDSS remained stable on a mild level of disability in one functional system during the prospective twelve-month period, indicating no disease progression on treatment with IM IFNb-1a. The average EDSS score was 1.9 ± 1.4 at Visit 1 (*n* = 205/235 evaluable), 1.9 ± 1.3 at Visit 2 (*n* = 160/168), 1.9 ± 1.3 at Visit 3 (*n* = 141/148), 1.9 ± 1.3 at Visit 4 (*n* = 122/129) and 1.9 ± 1.2 at Visit 5 (*n* = 102/107). Compared to the baseline visit, the EDSS had not changed after 12 months of IM IFNb-1a treatment (median difference: 0.0; range: −3.0 to 3.0; *n* = 99).

**Figure 5:**
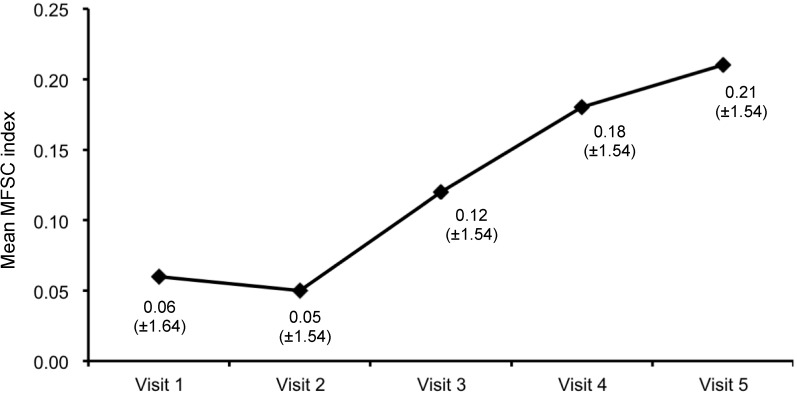
Course of mean Multiple Sclerosis Functional Composite (MSFC) index (±SD) during the prospective observational period on treatment with IM IFNb-1a (patients with documentation of the MSFC at all visits, *n* = 29).

For 29 of the 235 patients treated with IM IFNb-1a, complete data of the MSFC from all visits were available. Higher MSFC scores compared to baseline or prior measurements indicate neurologic improvement [11]. Among all evaluable patients, the mean MSFC composite score was relatively poor at baseline (−1.41 ± 3.27, *n* = 64), which was probably due to the low number of patients completing the whole test. The MSFC score was −0.36 ± 2.08 at Visit 2 (*n* = 48), −0.01 ± 1.59 at Visit 3 (*n* = 41), 0.03 ± 1.76 at Visit 4 (*n* = 33) and 0.16 ± 1.41 at Visit 5 (*n* = 34). Among subjects with complete data at all visits, the mean MSFC score steadily increased during the prospective observation period on treatment with IM IFNb-1a from 0.06 ± 1.64 at baseline to 0.21 ± 1.54 at Visit 5 (12 months, Figure 5), indicating a positive trend. The mean MSFC scores were 0.05 ± 1.54 at Visit 2, 0.12 ± 1.54 at Visit 3, and 0.18 ± 1.54 at Visit 4.

In the majority of evaluable patients, treatment with IM IFNb-1a remained unchanged from the previous visit at Visit 2 (*n* = 148/168, 88.1%), Visit 3 (*n* = 133/148, 89.9%), Visit 4 (*n* = 110/129, 85.3%) and Visit 5 (*n* = 99/107, 92.5%). Due to the low incidence of treatment modifications (discontinuation or interruption) and frequently missing information, the data on reasons for treatment discontinuation or interruption provide no reliable information.

Five serious adverse events were reported in four patients. Three adverse events were classified as serious because of hospitalization and two because of medical significance. The serious adverse events were depressive episode, lupus erythematosus, angioedema, urticaria and hip total endoprosthesis (due to congenital hip damage). Two of the serious adverse events, namely angioedema and urticaria in a 47-year-old woman, were assessed as causally related to the study medication. The patient had a history of urticaria due to GA and an allergic reaction to SC IFNb-1a. Treatment with IM IFNb-1a was permanently discontinued due to serious adverse events in three cases, transiently interrupted in one case, while information regarding the remaining case was missing. No other data on adverse events of IM IFNb-1a were reported.

## 3. Discussion

The AXIOM trial was an open-label, multicenter, observational study intended to reflect everyday clinical practice, which comprised a retrospective (3 or 12 months) and a 12-month prospective phase. A total of 235 patients with RRMS, who had not been treated with MS-medications for at least three months prior to enrolment, were included in the study and started treatment with IM IFNb-1a. The primary objective of the study was to evaluate the efficacy of newly-initiated IM IFNb-1a treatment in patients with RRMS after a treatment-free interval. The prospective data were compared with retrospective data on the course of disease and MS therapy.

The average duration of the treatment-free interval prior to initiation of prospectively-observed IM IFNb-1a ranged from 1.1 years (GA) to 2.8 years (IFNb-1a). Some patients were switched immediately or after a short treatment gap to IM IFNb-1a. The results of the AXIOM trial suggest that, at least to some extent, year-long treatment gaps or delayed initiation of treatment are part of clinical practice in Germany. On the other hand, the relatively slow recruitment of patients, which resulted in a smaller than planned sample size (235/500), may indicate that the majority of patients was either switched directly or after an only short delay.

The reasons for discontinuation or interruption of previous treatment differed between the DMTs in the AXIOM trial, which is consistent with the results of some, but not all other studies [12,13,14]. Injection fatigue, adverse reactions and injection-related side effects were frequent reasons for stopping previous DMTs; both latter reasons were less often reported for IM IFNb-1a than for the SC formulations. The low likelihood of treatment interruption due to injection site reactions associated with IM IFNb-1a is supported by other studies [12,14]. A lack of efficacy was a major reason to interrupt, discontinue or change DMT treatment in other studies [5,6,14,15,16], but reasons may vary depending on treatment duration [12]. The results of the AXIOM trial suggest that from a patient’s point of view, adverse events or injection reactions appear to be at least as important a reason to discontinue DMTs as lack of efficacy. As already suggested by Beer *et al.* [12], amongst other factors, injection site reactions should be considered when treatment decisions are made to increase the chance of optimal therapy in the long term.

Compared to preceding MS treatments, injection-related side effects, as indicated by the VAS, declined with IM IFNb-1a treatment. In several studies, the likelihood of injection site reactions was higher with SC DMTs than IM IFNb-1a, e.g., the randomized EVIDENCE trial comparing SC IFNb-1a 44 µg and IM IFNb-1a (83% *vs.* 28%) [12,17,18]. The patient self-assessed quality of life rated on a VAS improved on current treatment with IM IFNb-1a as compared to preceding MS treatments. The magnitude of improvement as rated by the patients (between 7.7 and 14.3 points on a 25-point VAS) would appear clinically meaningful, but the relevance of the observed changes for patients cannot be currently assessed. In accordance with the AXIOM trial, the majority of trials with IM IFNb-1a showed a trend towards better quality of life, particularly in the physical domains, or at least a stable quality of life [19,20,21].

The results of studies investigating the effect of switching DMTs on relapse rate are not totally consistent [15,22,23,24]. In the AXIOM trial, the relapse rate was markedly lower during prospective treatment with IM IFNb-1a (0.2 per quarter on average) compared to retrospective data reflecting the three-month period prior to enrolment (1.1), when the majority of patients did not receive DMTs. The observational, non-controlled AXIOM trial tends to support the results of most, but not all previous studies [2,3,17,25,26,27,28,29,30], suggesting that the disease course is worse when treatment initiation of DMTs is delayed or treatment is interrupted for longer periods of time.

The MSFC is a reliable and sensitive instrument for the assessment of the functional status of MS patients [11,31,32,33], but its interpretation may be complicated by training effects and the lack of a widely-accepted cut-off point of a clinically-meaningful change, despite some investigations to determine a clinically-reliable change [11,34,35,36,37,38]. Among participants of the AXIOM trial with complete MSFC data, the MSFC score steadily increased on prospectively-observed treatment with IM IFNb-1a, which is suggestive of an improving functional status. However, the improvement of the MSFC score and its relevance is difficult to interpret (e.g., small sample size and training effect) [32,35], and the potential clinical relevance of the change cannot be appropriately determined. Despite these uncertainties, disability did not progress on treatment with IM IFNb-1a, as evidenced by stable EDSS values, the still internationally most widely-used tool for the assessment of disability in MS [7,29].

Observational, non-interventional studies, such as AXIOM, which are conducted under “real-life” conditions, can extend the knowledge beyond the more restrictive conditions of randomized, controlled studies, but their limitations merit discussion. The AXIOM trial consisted of a retrospective and a prospective part, and particularly for the retrospective part, recall bias cannot be excluded. In the absence of a control group, the reduced relapse rate on prospective treatment with IM IFNb-1a may represent a regression to the mean phenomenon or may have been subject to other forms of bias.

Only about half of the planned number of patients (235/500) was included in the AXIOM trial, which might reflect a difficulty in recruiting patients who are not treated for at least three months. The low number of patients who did not fulfil the inclusion criterion (three-month treatment-free interval) is not presumed to represent a major drawback. Considering that the non-interventional AXIOM trial was aimed to mirror “real-life” conditions, this finding rather indicates that direct switches of MS-specific therapy, as well as treatment breaks are part of the usual clinical practice. Some of the endpoints were optional, and the low number of data available for some of the endpoints limits the reliability of these results. This notwithstanding, e.g., for the MSFC, the high number of missing values is informative, as it at least indicates that this (time-consuming) assessment tool is not in common use in Germany. The data are only representative for a cohort of mildly-disabled RRMS patients, and the characteristics of patients who start treatment with a delay or take treatment breaks of variable length may differ from those with no or only short treatment gaps. Furthermore, the inclusion of patients starting treatment with a pre-specified IFNb formulation may have introduced a selection bias. Lastly, the *post hoc* statistical analyses (ANOVA) should be interpreted with caution.

## 4. Methods

### 4.1. Study Design and Objectives

The AXIOM study was an open-label, multicenter, non-interventional, observational study in Germany consisting of a 3- to 12-month retrospective and a 12-month prospective part. The study was approved by the independent Ethics Commission of the Baden-Wuerttemberg Medical Association before initiation (F-2010-055, 20 July 2010). The study was performed in accordance with the ethical standards laid down in the 1964 Declaration of Helsinki and amendments. All patients provided written informed consent prior to enrolment. The requirements for data protection of the individual patient were fulfilled, because all data were pseudonymized (*i.e.*, each patient’s name was substituted by a unique code). The first patient was enrolled on 5 July 2010, and the last patient completed the study on 18 January 2012. The study was funded by Biogen Idec, Ismaning, Germany.

The primary objective of the AXIOM study was to evaluate the efficacy (relapse rate and disability progression) over 12 months of newly-initiated treatment with IM IFNb-1a after a treatment-free interval of at least three months under usual practice conditions. The subdivision of the study into a retrospective and prospective part allowed for an assessment of the disease course over different stages of treatment or no treatment. Particular attention was paid to the previous disease course, any MS treatments and treatment characteristics (change or interruption of treatment, treatment efficacy and reasons for stopping previous treatment) during the last 3 to 12 months (retrospective).

### 4.2. Subjects

Patients with RRMS, who had a treatment-free interval of at least three months prior to inclusion and for whom the decision to initiate treatment with IM IFNb-1a was made by the treating physician before inclusion could participate in the study. No particular exclusion criteria were defined.

### 4.3. Treatment

The patients were treated with IM IFNb-1a under the conditions specified in the Summary of Product Characteristics (30 µg once weekly IM). The planned treatment duration was 12 months.

### 4.4. Assessment and Documentation

Data were collected at Visits 1 (baseline), 2, 3, 4 and 5 (*i.e.*, 3, 6, 9 and 12 months after inclusion). At each evaluation time point, the participating physicians completed standardized online questionnaires, which were complemented by patient self-reports. The following data were collected at Visit 1 (retrospective with respect to treatment and disease history): demographic data (gender, age, height, body weight), MS anamnesis, including first symptoms of MS, MS treatment history, including reasons for discontinuation of previous treatment, Expanded Disability Status Scale (EDSS) [39], relapse rate in the last 3 or 12 months, including steroid-treated relapses, and MRI (optional) before study entry. The EDSS, Multiple Sclerosis Functional Composite score (MSFC, optional) and MRI (optional) were documented at all scheduled visits. At Visits 2, 3, 4 and 5, the number of relapses, including steroid-treated relapses, and the status of treatment with IM IFNb-1a (continued, interrupted or discontinued and the reasons for interruption or discontinuation) in comparison to the previous visit were reported. At Visit 3, the degree of injection fatigue, measured by means of a Visual Analog Scale (VAS), injection reactions, flu-like symptoms and quality of life on treatment with IM IFNb-1a in comparison to the previous MS treatment was rated by the patients, if applicable. The patients were asked to indicate the magnitude of discomfort by recall on a VAS. The undivided Visual Analog Scale had a range of “none” (=1) to “very intense” (=25) and emoticons representing poorest possible (=1) and best possible quality of life (=25). The VAS is a generic, *i.e.*, not disease-specific, instrument commonly used to assess health status and is, for example, part of the EuroQol-5D (EQ-5D) health questionnaire [40], but the VAS used in the AXIOM trial differs from that of the EQ-5D (vertical, scaled line). A 100-mm horizontal VAS had been used for the assessment of injection pain of a novel GA formulation and a novel IFNb injection device [41,42].

The primary endpoint was the efficacy of IM IFNb-1a as assessed by EDSS [39] and relapse rate. We compared data obtained during the prospective twelve-month period to retrospective data at 3 or 12 months prior to study enrolment. Secondary efficacy parameters were the MSFC (optional), a validated multidimensional composite measure of function developed by the National MS Society’s (NMSS) Clinical Outcomes Assessment Task Force [34,43,44,45] and MRI (optional). The MSFC has high test-retest reliability, and the construct, concurrent and criterion validity of the MSFC has been shown [11,31,34,46]. For the assessment of functional impairment by the MSFC, *Z*-scores were calculated for each of the three tests (function of legs and ambulation (timed 25-foot walk), function of arms and hands (9-hole peg test) and cognitive function (Paced Auditory Serial Addition Test 3 (PASAT 3), second version)) [11,32], which represent the difference between the test and a reference value. The MSFC index was computed from the *Z*-scores of each component. For the calculation of the MSFC index value, the reference values in the database of the NMSS were used [34,44]. Patient instructions for the components of MSFC are available in German [44].

Serious adverse events had to be reported online or on a paper report form.

### 4.5. Statistical Analyses

A total of 500 subjects was planned for inclusion, but no formal sample size calculation was performed. No imputation was used to replace missing values.

The data were analyzed by means of descriptive statistics with the computer program Statistical Analysis System (SAS) for Windows. For certain parameters, *post hoc* statistical analyses were performed by using analysis of variance (ANOVA). Data are expressed as the mean ± standard deviation (SD), unless otherwise indicated.

## 5. Conclusions

Despite the recommendation to initiate treatment as early as possible after definite diagnosis of MS with active, relapsing disease [47], late initiation of DMTs and/or long treatment gaps appear to be part of clinical practice in the treatment of RRMS in Germany. Taking into account the limitations described above, the results of the AXIOM study indicate that treatment gaps adversely affect the disease course. IM IFNb-1a appears to be effective at reducing relapses and stabilizing the EDSS in RRMS patients after a treatment-free interval. The participants of the AXIOM trial consistently expressed more positive opinions on the severity of injection fatigue, injection reactions and flu-like reactions (except GA), as well as quality of life during treatment with IM IFNb-1a than during treatment with previous DMTs. These results may be useful in optimizing MS treatment in everyday clinical practice and suggest that IM IFNb-1a is an effective and well-accepted treatment option in currently untreated patients or those who are dissatisfied with their previous or current DMT regimen. To improve persistence and to increase the chances of optimal long-term treatment benefit, a timely treatment modification may not only be considered in the case of non-response, but also in patients who experience serious problems with their DMT injection regime.
